# NOMePlot: analysis of DNA methylation and nucleosome occupancy at the single molecule

**DOI:** 10.1038/s41598-019-44597-2

**Published:** 2019-05-31

**Authors:** Francisco Requena, Helena G. Asenjo, Guillermo Barturen, Jordi Martorell-Marugán, Pedro Carmona-Sáez, David Landeira

**Affiliations:** 1 Centre for Genomics and Oncological Research (GENYO), Avenue de la Ilustración 114, 18016 Granada, Spain; 2 Department of Biochemistry and Molecular Biology II, Faculty of Pharmacy, University of Granada, Granada, Spain

**Keywords:** Software, Embryonic stem cells, DNA methylation, Nucleosomes

## Abstract

Recent technical advances highlight that to understand mammalian development and human disease we need to consider transcriptional and epigenetic cell-to-cell differences within cell populations. This is particularly important in key areas of biomedicine like stem cell differentiation and intratumor heterogeneity. The recently developed nucleosome occupancy and methylome (NOMe) assay facilitates the simultaneous study of DNA methylation and nucleosome positioning on the same DNA strand. NOMe-treated DNA can be sequenced by sanger (NOMe-PCR) or high throughput approaches (NOMe-seq). NOMe-PCR provides information for a single locus at the single molecule while NOMe-seq delivers genome-wide data that is usually interrogated to obtain population-averaged measures. Here, we have developed a bioinformatic tool that allow us to easily obtain locus-specific information at the single molecule using genome-wide NOMe-seq datasets obtained from bulk populations. We have used NOMePlot to study mouse embryonic stem cells and found that polycomb-repressed bivalent gene promoters coexist in two different epigenetic states, as defined by the nucleosome binding pattern detected around their transcriptional start site.

## Introduction

Eukaryotic DNA is wrapped around histones octamers to form nucleosomes and chromatin^1^. Nucleosomes and chromatin are major regulators of gene transcription which is a highly dynamic process and it is controlled by the coordinated action of a large set of transcription factors and chromatin regulators. Precise temporal and spatial control of gene regulation is critical for mammalian development and correct tissue homeostasis. Importantly, different cells within the same population can show distinct transcriptional and epigenetic profiles. This is particularly relevant in the context of stem cell differentiation and cancer progression where cell-cell differences can have critical functional consequences^2,3^. Thus, great efforts are currently being made to develop methods to measure transcriptional and epigenetic cell-cell differences with the determination of providing tools that will reveal key insights to better understand human development and disease^4^.

Chromatin accessibility and nucleosome positioning are two major regulators of the eukaryotic genome. These have been traditionally assayed by sequencing DNA upon treatment with nucleases that are sensitive to chromatin compaction and nucleosome binding. Treatment with DNase I or micrococcal nuclease followed by high throughput sequencing (DNase-seq or MNase-seq) facilitates the identification of regulatory elements and nucleosome positioning respectively^5,6^. More recently the assay for transposase-accessible chromatin followed by sequencing (ATAC-seq) has been proved to be useful to measure chromatin accessibility and nucleosome binding^7^. The initial version of these three methods require thousands of cells as input material and their analysis includes a step to average sequencing reads – thus averaging out cell-cell differences within the analysed population. Recently, these protocols have been adapted to allow single cell analysis of chromatin accessibility and nucleosome binding genome-wide^8–11^.

The nucleosome occupancy and methylome assay (NOMe-assay) is a recently developed method that is based on the use of the prokaryotic methyltransferase M.CviPI^12,13^. M.CviPI methylates cytosines in GpC sites within regions of DNA that are not occupied by nucleosomes. Because M.CviPI methylation is specific for GpC sites, endogenous DNA methylation at CpG sites is preserved. After standard bisulphite conversion of M.CviPI-treated chromatin, non-methylated cytosines will be converted into uracil and will be read as thymine upon DNA sequencing, revealing the regions of DNA that were nucleosome bound and not accessible by M.CviPI. NOMe-treated DNA can be sequenced upon PCR amplification using traditional sanger (NOMe-PCR, to analyse candidate regions)^12^ or after library construction using high throughput sequencing (NOMe-seq, genome-wide analysis)^13^. In both cases the resulting sequences will retain information of endogenous DNA methylation and nucleosome binding for the same molecule of DNA.

NOMe-PCR has been useful to analyse candidate regions at the single molecule using lollipop graphics^12,14^. This type of analysis is very convenient for functional studies focused in one or few regions of interest. The main drawbacks are that the analysed region is limited by the length of the PCR product and that the bioinformatic analysis is time consuming because it has not been automatized. On the other hand, NOMe-seq provides genome-wide data and current tools allow to obtain population-averaged measures^13,15^. Recently, adaptations of NOMe-seq to use single cells as starting material have been reported (scNOMe-seq)^16^. However, it is not strictly required to use single cell as input to carry out genome-wide analysis at the single molecule in NOMe assays. Given the nature of the technic, NOMe-seq datasets obtained from bulk populations enclose single molecule information for all the genome. We decided to develop a bioinformatic tool that would allow us to interrogate these datasets and obtain a cost-effective method of extracting precious information at the single molecule for any region of the genome. As a use example we analysed the epigenome of mouse embryonic stem cells (mESCs).

Pluripotent mESCs are derived from the developing blastocyst and are a transcriptionally heterogenous population of cells that retain the ability to differentiate into the three germ layers^17,18^. In mESCs, hundreds of developmentally regulated genes are repressed by polycomb repressive complexes (PRCs)^19^ and show opposing histones marks associated with both active and inactive transcription - hence their designation as bivalent genes^20^. Bivalent genes are bound by poised RNA-Polymerase II which is believed to prime these genes for coordinated activation during cell differentiation^21^. The molecular mechanism by which PRCs repress transcription at bivalent genes remains poorly understood.

Here, we describe the development of a web tool (NOMePlot) to analyse NOMe-PCR and NOMe-seq data. NOMePlot provides a complementary and cost-effective alternative to scNOMePlot. NOMePlot facilitates analysis and representation of DNA methylation and nucleosome positioning in single DNA molecules from any genomic region using as input NOMe-seq datasets obtained from bulk populations. NOMePlot is freely available and it is designed to be used by molecular biologists without bioinformatic skills. NOMePlot also supports analysis of traditional bisulfite-treated-DNA-sequencing (BS-seq) datasets. To demonstrate the usefulness of the application we have generated and analysed NOMe-seq datasets for mouse embryonic stem cells (mESCs) and found the existence of distinct subpopulations of molecules with different nucleosome arrangements at bivalent but not at silent DNA-methylated gene promoters.

## Results

### Development of NOMePlot

**Figure 1:**
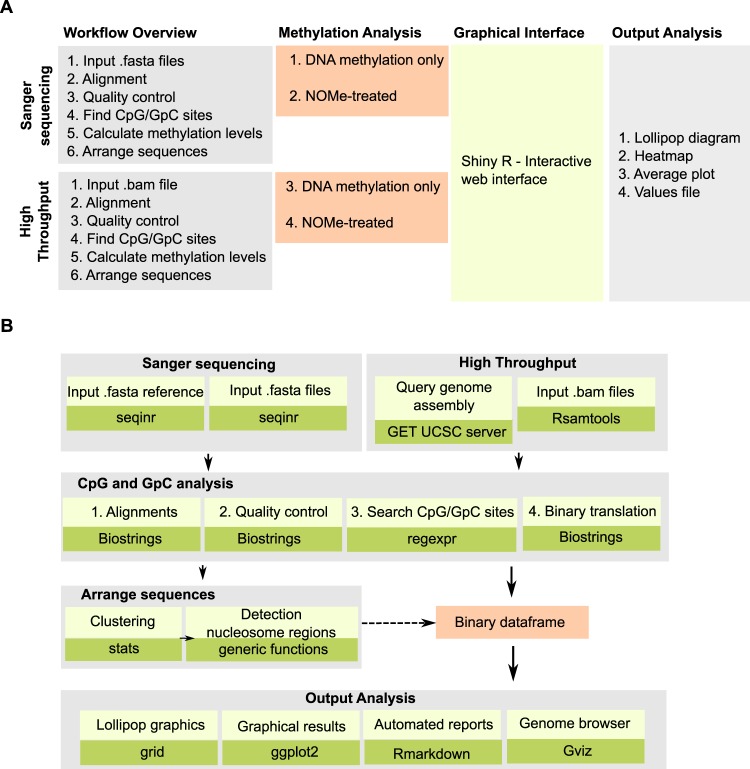
Schematic design overview of NOMePlot. (**A**) Scheme showing key aspects and functionalities of NOMePlot. (**B**) Flow diagram highlighting the main R packages used for the development of NOMePlot.

Currently, there are tools to analyse BS-treated DNA sequenced by Sanger (i.e. BiQ Analyzer^22^ or MethVisual^23^) but there is no software to automatically analyse NOMe-PCR data. Likewise, there is availability of tools to analyse average nucleosome positioning using NOMe-seq^13^ and other technics^24^, but there is not a software that facilitates the analysis at the single molecule of BS-seq and NOMe-seq datasets generated by high throughput sequencing. Thus, we set to develop a bioinformatic tool to carry out single molecule analysis of nucleosome binding and DNA methylation using NOMe-PCR and NOMe-seq datasets as input data. To make NOMePlot more amenable to the front-end user, it was design as an interactive web application that can be effectively run by any molecular biologist with no technical expertise in the field of bioinformatics. Analysis of NOMe data is based on the activity of the GpC specific methyltransferase M.CviPI followed by bisulfite (BS) treatment. Thus, generated datasets are bioinformatically similar to the ones generated during traditional analysis of DNA methylation, where DNA is also treated with BS. To make NOMePlot of wider application, it was designed in four different modules that are integrated though a graphical interface (Fig. 1A) and it offers analysis of NOMe and BS-treated only data. The first two modules provide tools to analyse sequences obtained by traditional Sanger technology, and the other two are designed to interrogate BS-seq and NOMe-seq datasets generated by high throughput sequencing (Figs 1A, S1A,B).

NOMePlot is written by combining function from publicly available R packages and custom scripts (Fig. 1B). The graphical user-interface is developed in Shiny R which is also an R package that facilitates to build interactive web applications (http://shiny.rstudio.com/). In the module designed for Sanger analysis, sequences must be provided in standard fasta format to be aligned (seqinr), quality controlled (Biostrings), CpGs and GpCs found (regexpr) and presence of methylation determined (biostrings). Sequences are then clustered (stats) and nucleosomes marked (generic functions). Thereafter, different packages are used to browse across the genome (Gviz), produce the reports (Rmarkdown) and generate the Lollipop graphics (grid) or other graphical results including plots and heatmaps (ggplot2) (Fig. 1B). Sequences generated by high-throughput sequencing for a number of organisms including human, mouse and yeast can be introduced using files in the standard bam format. Once the user sets the target genomic region to analyse, NOMePlot will connect to the University of California and Santa Cruz (UCSC) server (GET UCSC server) to download the reference  sequence. NOMePlot will transform input sequences into fasta format (Rsamtools) and then perform the analysis using the same pipeline implemented to analyse sanger sequences (Fig. 1B).

One of the most useful aspects of NOMePlot is that it can produce lollipop graphics analysis for NOMe-PCR and NOMe-seq data. This implementation is based on the following principles; (1) cytosines in CpGs dinucleotides are represented as coloured circles depending on whether they were methylated (black) or not (white). In the underneath panel, cytosines in GpCs dinucleotides are represented as white if they were nucleosome protected and coloured in blue if they were accessible and methylated by M.CviPI. (2) Each sequence is plotted in one line and columns correspond to individual CpG or GpC position. (3) The generated lollipop graphic is scaled taking into account the relative distance of CpGs and GpCs in the genome. (4) NOMePlot provides tools to find optimal clustering of sequences by controlling the “clustering variable” (CpG or GpC) and the “clustering window” parameters (20 to 160 bps, 60 is optimal is most cases). “Clustering window” calculates the percentage of methylation for any given position in the selected nucleotide window (for example, methylation value at CpG in position 200 bp will be the average in all CpGs found from 130 bp to 270 bp if a 140 nt windows is chosen), builds a matrix with this value and arrange sequences with most similar patterns. (5) To facilitate analysis of nucleosome binding, NOMePlot allows to establish “Region size length (100–160 bps)” and “stringency (0.5 to 1)” to mark with solid lines regions that show consistent protection and can be inferred to be nucleosome bound. For example, size length of 140 and stringency of 0.8 will draw solid lines in regions of 140 bp where there are more than 80% of white consecutive GpC. (6) NOMePlot also permits to mark nucleosome positions, label TSS and choose the colour to represent cytosine methylation.

Importantly, output analysis files are optimized for scientific presentation and publication (see methods) including the lollipop graphic schemes which can be easily downloaded as a vector graphic.

### Automatic analysis of candidate regions using NOMe-PCR

**Figure 2:**
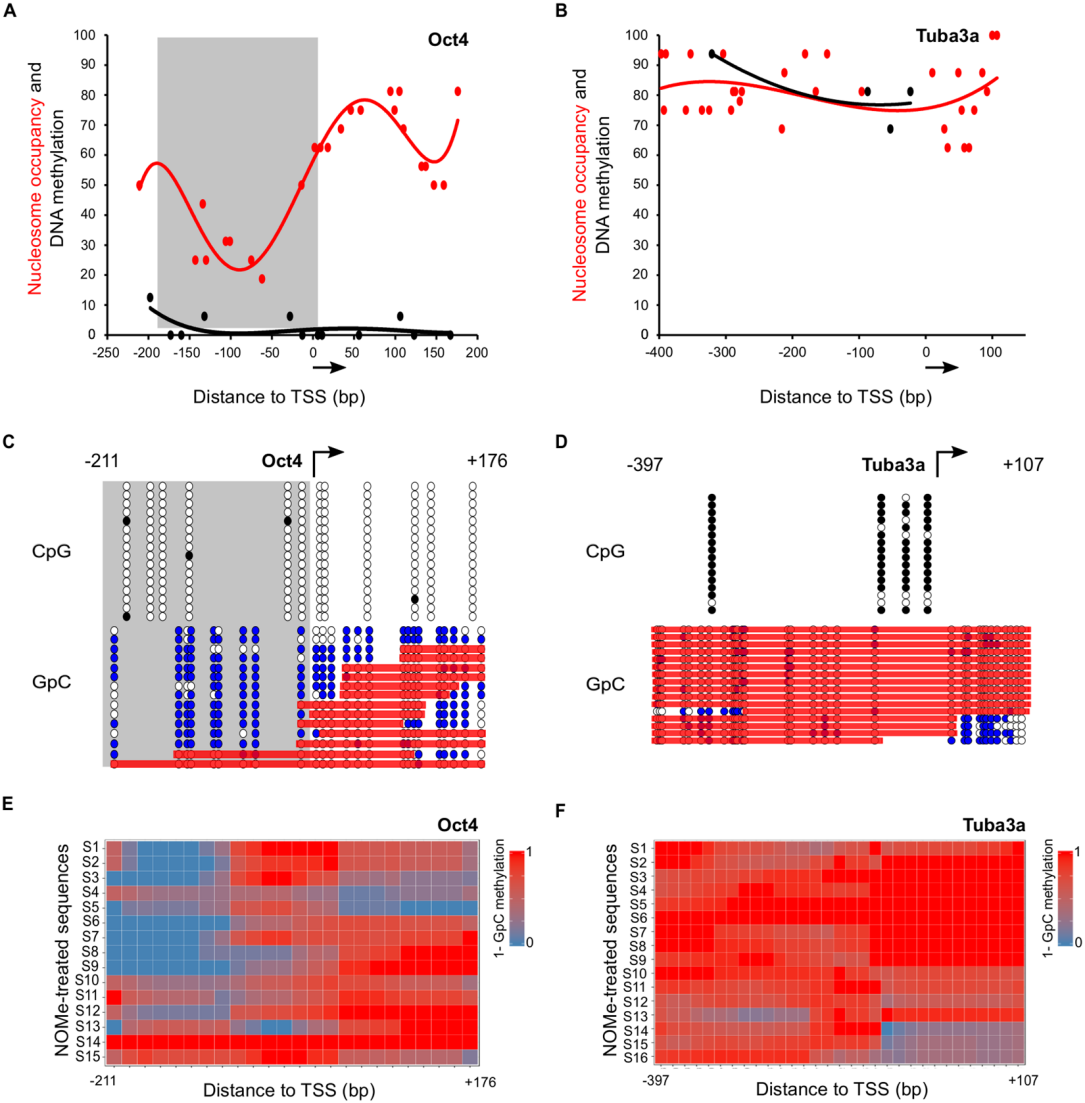
Analysis of *Oct4* and *Tuba3a* gene promoters using NOMe-PCR followed by NOMePlot in mESCs. (**A**,**B**) Scatter plots with trendline showing population-averaged nucleosome occupancy (red) and DNA methylation (black) relative to the TSS of an active gene (*Oct4)* (**A**) and a DNA methylated silent gene (*Tuba3a*) (**B**). Nucleosome occupancy is calculated as the percentage of unmethylated GpC sites. DNA methylation is plotted as the percentage of methylated CpG dinucleotides. Arrow indicates the direction of transcription. (**C**,**D**) Lollipop diagram showing DNA methylation and nucleosome occupancy at the single molecule for *Oct4* (**C**) and *Tuba3a* (**D**). Top panels show cytosine methylation at CpGs (white for unmethylated, black for methylated) and bottom panels show methylation patterns at GpC dinucleotides (white for unmethylated, blue for methylated). Each line represents one molecule and each row correspond to a genomic position around the TSS (arrow). Red bars highlight GpC unmethylated regions long enough to accommodate a nucleosome. NDRs are marked with a grey box. (**E,F**) Heatmap of nucleosome occupancy for *Oct4* (**E**) and *Tuba3a* (**F**) upon clustering of fifteen and sixteen sequences respectively. Nucleosome occupancy was calculated for each position around the TSS (X axis) as the average value of unmethylated cytosines at GpCs found within 140 bp windows. Color code goes from red (100% occupancy, 1-GpG = 1) to blue (0% occupancy, 1-GpC = 0).

As use case we analysed nucleosome binding and DNA methylation in mESCs using NOMe-PCR and NOMe-seq. First, we carried out NOMe-PCR for candidate gene promoters that are known to be active (*Oct4, Actb)* or known to be inactive and heavily methylated (*Tuba3a, Myf5*) in mESCs^25^. Analyses of population-averaged profiles of DNA methylation and nucleosome occupancy show that the promoters of *Oct4* (Figs 2A, S2A, black line) and *Actb* (Figure S2C, black line) are not methylated and both *Oct4* (Figs 2A, S2A, red line) and *Actb* (Figure S2C, red line) present a nucleosome depleted region (NDR) around the transcriptional start site (TSS) - as expected for a highly expressed gene. In contrast, the promoters of DNA methylated silent genes like *Tuba3a* (Figs 2B, S2B) and *Myf5* (Figure S2F) display expected high level of DNA methylation and nucleosome occupancy around the promoter region with no detectable NDR.

Analysis of *Oct4* and *Actb* active promoters using lollipop graphics showed consistent absence of DNA methylation at CpGs in individual sequences as expected (Figs 2C, S2D, top panel). In agreement with the population averaged profile, most sequences (thirteen out of fifteen sequences for *Oct4* and eight out of twelve sequences for *Actb*) show a well-established NDR upstream of the TSS in these active genes (Figs 2C, S2D, bottom panel, grey box). In contrast, single molecule analysis of *Tuba3a* and *Myf5* gene promoters confirmed consistent DNA methylation (Figs 2D, S2G, top panel) in the large majority of analysed sequences (fifteen for *Tuba3a* and thirteen for *Myf5*). In fitting with transcriptional silencing and DNA methylation at the promoters of these genes, nucleosome binding upstream of the TSS was detected in all sixteen sequences analysed for *Tuba3a* (Fig. 2D, bottom panel) and in twelve out of thirteen for *Myf5* (Figure S2G, bottom panel).

NOMePlot can also produce a heatmap of clustered sequences considering average methylation values for a given nucleotide window. Regions with consistent accessible GpCs are represented in blue while non-accessible ones are plotted in red. Heatmaps analysis of *Oct4, Actb, Tuba3a and Myf5* promoter regions using 140 bp nucleotide windows showed very similar results to the lollipop analysis. *Oct4 and Actb* promoter regions (Figs 2E, S2E) show an NDR in most cells of the population while *Tuba3a* and *Myf5* (Figs 2F, S2H) promoters are homogeneously bound by nucleosomes around the TSS. Taken together these analyses demonstrate the utility of NOMePlot to determine DNA methylation and nucleosome binding at the single molecule.

### Single molecule analysis of DNA methylation and nucleosome binding using NOMe-seq datasets

**Figure 3:**
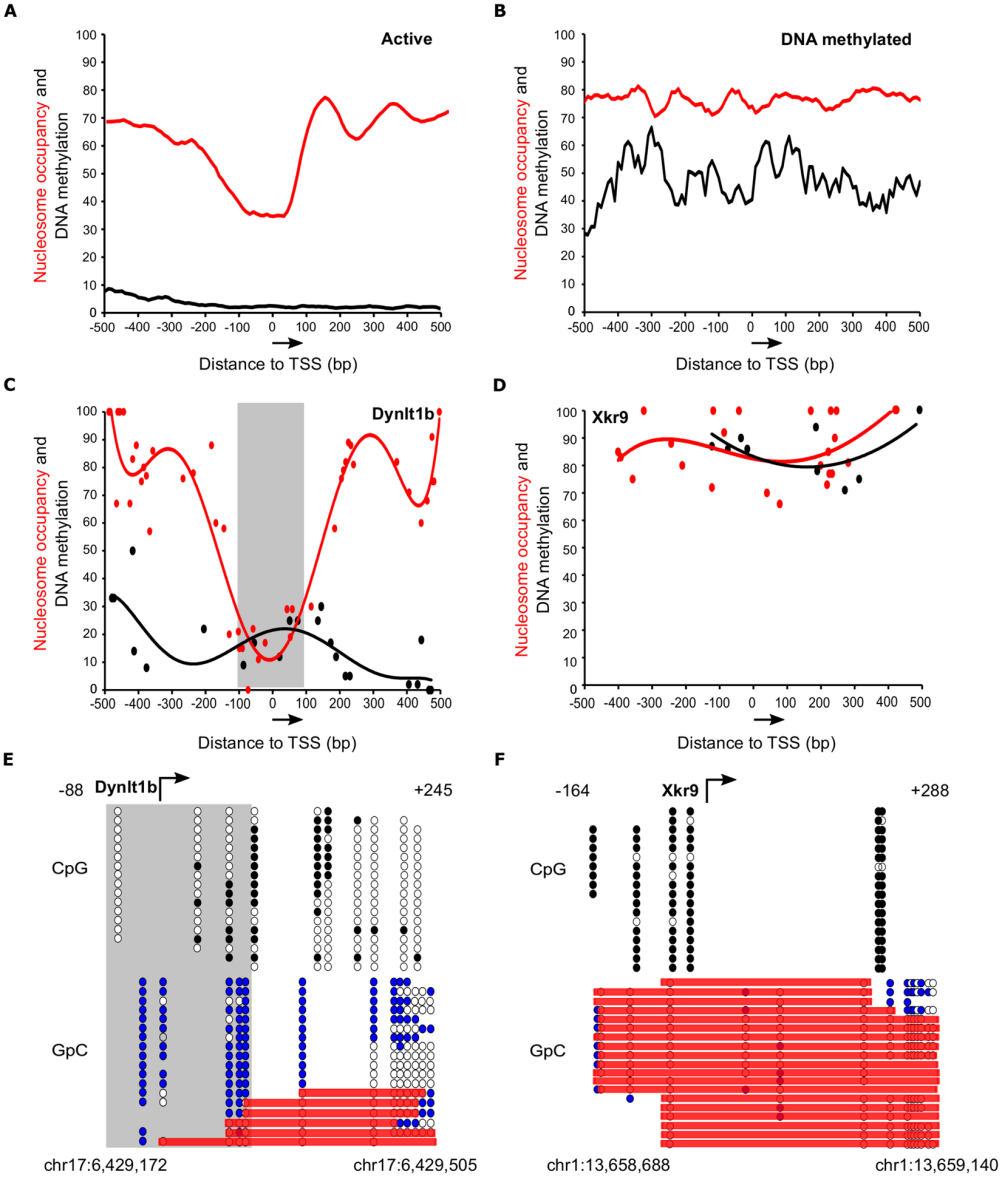
Single molecule analysis of *Dynlt1b* and *Xkr9* gene promoters using genome-wide NOMe-seq followed by NOMePlot in mESCs. (**A,B**) Population-averaged plots showing nucleosome occupancy (red, percentage of unmethylated GpCs) and DNA methylation (black, percentage of methylated CpGs) relative to the TSS (±500 bp) for transcriptionally active genes (n = 2512) (**A**) and DNA methylated silent genes (n = 1080) (**B**) in mESCs. Arrow indicates the direction of transcription. (**C,D**) Scatter plots with trendline showing population-averaged nucleosome occupancy (red) and DNA methylation (black) relative to the TSS of an active gene (*Dynlt1b)* (**C**) and a DNA methylated silent gene (*Xkr9*) (**D**). Nucleosome occupancy is calculated as the percentage of unmethylated GpC sites. DNA methylation is plotted as the percentage of methylated CpG dinucleotides. Arrow indicates the direction of transcription. (**E**,**F**) Lollipop diagram showing DNA methylation and nucleosome occupancy at the single molecule for *Dynlt1b* (**E**) and *Xkr9* (**F**). Top panels show cytosine methylation at CpGs (white for unmethylated, black for methylated) and bottom panels show methylation patterns at GpC dinucleotides (white for unmethylated, blue for methylated). Red bars highlight GpC unmethylated regions long enough to accommodate a nucleosome. NDRs are marked with a grey box. Genomic coordinates of the analyzed regions are indicated.

We carried out NOMe-seq using an established protocol^13^ and Illumina sequencing in a bulk population of wild-type mESCs. Comparison of population-averaged nucleosome occupancy (red line) and DNA methylation (black line) profiles for transcriptionally active (n = 2512, Fig. 3A) and inactive genes (n = 1080, Fig. 3B) confirmed that active genes are hypomethylated as compared to inactive ones (compare black lines in Fig. 3A,B). We could also confirm the presence of a NDR at the TSS followed by two phasic nucleosomes in active genes (Fig. 3A, red line) that is absent in silent genes (Fig. 3B, red line). Next, we generated a bam file containing mapped Illumina reads for a region of interest and we used NOMePlot to analyse candidate genes with high sequencing coverage. In agreement with the population-averaged plot (Fig. 3A,B), analysis of the active promoter *Dynlt1b* showed low DNA methylation at the promoter region and a very well established NDR at the TSS (Figs 3C, S3A) while at the transcriptionally inactive promoter of the *Xkr9 gene*, high levels of DNA methylation and nucleosome binding were found (Figs 3D, S3B).

Single molecule analysis of candidate promoters using lollipop graphics confirmed the existence of a NDR around the TSS of the active *Dynlt1b* promoter in seventeen out of eighteen sequences (Fig. 3E, bottom panel). In contrast, the promoter region of the transcriptionally inactive *Xkr9* showed consistent high levels of DNA methylation and nucleosome binding upstream of the TSS for all analysed sequences (Fig. 3F). Similar results were obtained for other active (*Prdx2*) and silent (*Dux*) gene promoters analysed (not shown). These observations are in fitting with our previous analysis of *Oct4*, *Actb*, *Myf5* and *Tuba3a* promoters using NOMe-PCR (Fig. 2) and show that NOMePlot can be used to study DNA methylation and nucleosome binding at the single molecule using NOMe-seq datasets from bulk populations.

### Cell-cell variability of nucleosome binding at bivalent promoters in mouse embryonic stem cells

**Figure 4:**
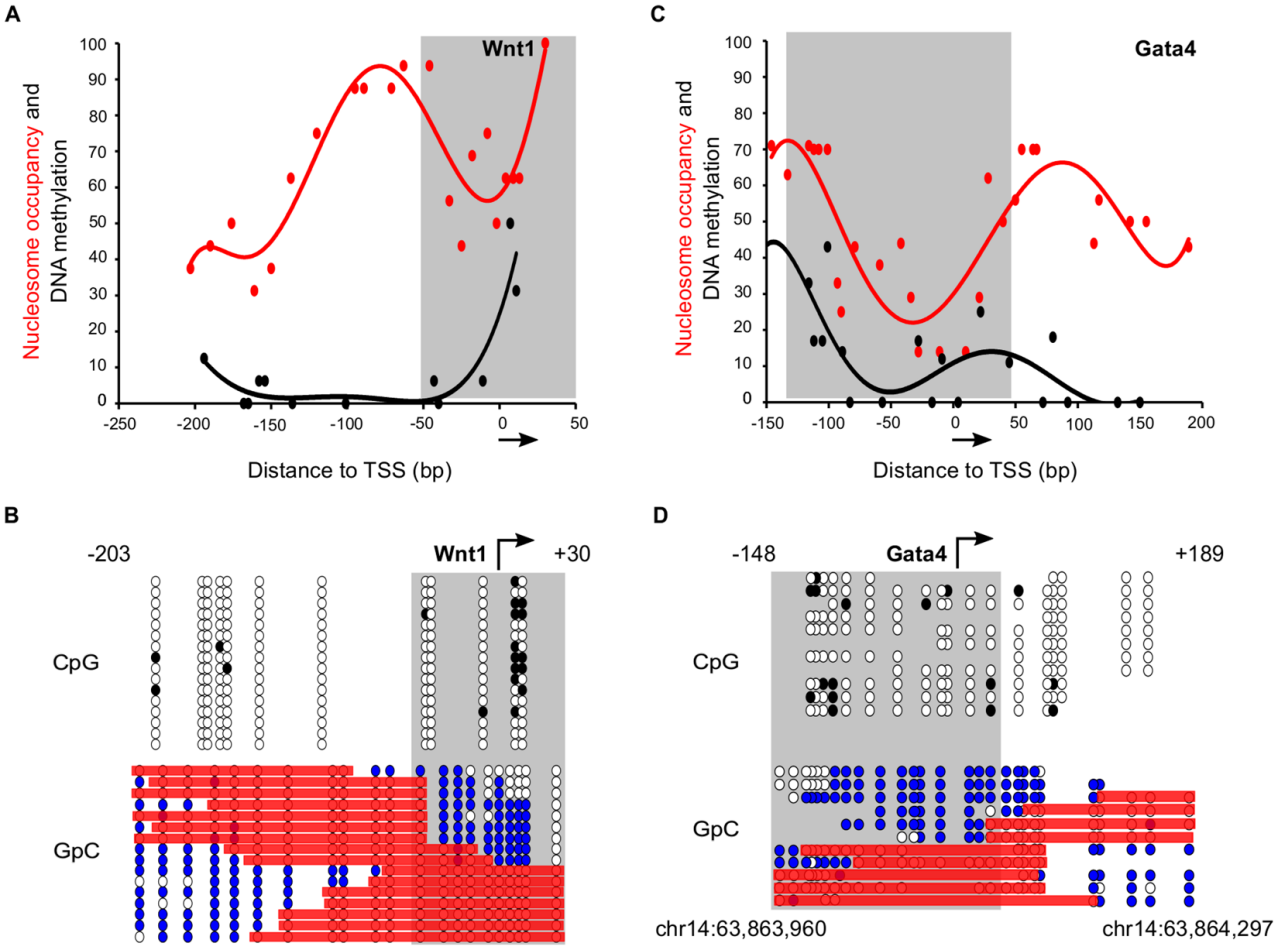
Single molecule analysis of nucleosome binding at bivalent promoters reveal alternative configurations within mESCs cell population. (**A**) Scatter plot with trendline showing population-averaged nucleosome occupancy (red) and DNA methylation (black) relative to the TSS of a bivalent gene (*Wnt1)* analyzed by NOMe-PCR. Nucleosome occupancy is calculated as the percentage of unmethylated GpC sites. DNA methylation is plotted as the percentage of methylated CpG dinucleotides. Arrow indicates the direction of transcription. (**B**) Lollipop diagram showing DNA methylation and nucleosome occupancy at the single molecule for *Wnt1* promoter region. Top panels show cytosine methylation at CpGs (white for unmethylated, black for methylated) and bottom panels show methylation patterns at GpC dinucleotides (white for unmethylated, blue for methylated). Red bars highlight GpC unmethylated regions long enough to accommodate a nucleosome. NDRs are marked with a grey box. (**C**) Scatter plot with trendline showing population-averaged nucleosome occupancy (red) and DNA methylation (black) relative to the TSS of the bivalent gene *Gata4* analyzed by NOMe-seq. Arrow indicates the direction of transcription. (**D**) Lollipop diagram showing DNA methylation and nucleosome occupancy at the single molecule for *Gata4* promoter region. Genomic coordinates are indicated in the analysis of Gata4 by NOMe-seq in (**D**) but not in analysis of *Wnt1* by NOMe-PCR in (**B**).

To shed some light onto how polycomb regulates chromatin accessibility and nucleosome binding in mESCs we focused our analysis on bivalent promoters. We used NOMePlot to analyse a representative subset of well-characterized^25^ bivalent gene promoters (*Wnt1*, *Pax3*, *Msx1*, *Sox7* and *Gata4*) using NOMe-PCR and NOMe-seq datasets. Population-averaged analysis of these promoter regions showed expected very low DNA methylation (black lines) levels. Importantly, the average nucleosome occupancy signal (red lines) around the TSS (Figs 4A,C and S4A,C,E, grey boxes) suggest the presence of an NDR at these promoters in agreement with previous studies^26,27^. Interestingly the average nucleosome occupancy signals around bivalent TSS are not as soundly defined as in active genes and usually range between 30 and 50% occupancy at the minimum of the NDR (Figs 4A,C and S4A,C,E).

Single molecule analysis of bivalent promoters revealed marked heterogeneity in nucleosome disposition for all examined genes. For example, at *Wnt1* promoter a clear NDR can be detected in nine out of sixteen sequences (56%) (Fig. 4B). In the remaining molecules, we consistently detect a nucleosome bound to this region that is occluding the NDR at this promoter. Similarly, for *Gata4* promoter (NOMe-seq dataset) six out of eleven sequences (54%) showed an NDR upstream of the TSS while the rest of sequences showed a well-positioned nucleosome in this region (Fig. 4D). Similar results were obtained for the rest of bivalent genes analysed such as *Pax3* (NDR in 50% of sequences), *Msx1* (NDR in 80% of sequences) and *Sox7* (NDR in 50% of sequences) (Figure S4B,D,F). This result suggests that the promoter of bivalent genes is accessible only in a fraction of molecules within the cell population in a given time. This observation might have important functional implications to understand epigenetic plasticity in pluripotent cells, because it suggests the existence of two alternative promoter configurations at polycomb target genes.

## Discussion

Analysis of gene regulation at single cell level is key to understand development and human disease. Therein, methods like RNA-seq, DNase-seq, ATAC-seq, MNase-seq, ChIP-seq and BS-seq are being adapted to function with single cells as input material^8–10,28–30^. Here we report the development of NOMePlot as an alternative method to carry out single molecule analysis of nucleosome binding and DNA methylation. Analysis of nucleosome binding at the single molecule from genome-wide datasets of bulk populations is only possible in NOMe-seq, because accessibility is encoded in the sequence and not in read counts.

Single-cell approaches to measure nucleosome binding are technically challenging and economically demanding. NOMePlot offers a cost-effective alternative to study nucleosome binding with resolution at the single molecule. It is freely available under the GNU license and it is designed to be used by molecular biologists without bioinformatic skills. It is particularly useful to complement genome-wide NOMe-seq and BS-seq studies by extracting locus-specific single-molecule analysis. The most important limitation of NOMePlot is the length of the region for which we have contiguous single-molecule information. In this study we have analysed Illumina 300 bp reads but NOMe-seq and NOMePlot can be coupled to sequencing platforms like Pac-Bio, increasing the average length of the reads by more than ten-fold and thus providing contiguous single molecule analysis along several kilobases. NOMe-assays deliver information of DNA methylation and nucleosome binding for the same molecule and thus, NOMePlot is particularly advantageous to examine links between DNA methylation and nucleosome positioning. Complementarily, NOMePlot can also handle BS-seq datasets allowing a large number of users interested in DNA methylation to easily complement their genome-wide studies with single cell analysis. Importantly, bisulphite treatment, and as a consequence NOMePlot, does not distinguish between 5-methylcytosine (5 mC) and 5-hydroxymethylcytosine (5 hmC). If this discrimination is required, NOMePlot could be used to analyse oxidative bisulfite sequencing (oxBS-seq) datasets^31^.

In this study we have used NOMePlot to analyse nucleosome binding at polycomb-target bivalent promoters in mESCs. Current literature suggests that PRC1 reduces chromatin accessibility of target promoters^32–35^, however, this is contradictory with reports showing that RNAPII can access and bind to PRC1-bound-promoters in ESCs^21^. Our analyses show cell-cell differences in nucleosome binding maps at bivalent genes in mESCs. This in contrast with transcriptionally active genes or heavily DNA methylated ones where we found more consistent nucleosome occupancy at the promoter regions across the population. We find that bivalent genes can exist in two alternative epigenetic configurations. The first one shows a clear NDR upstream of the TSS that could be potentially bound by RNAPII. In the second configuration, this region of DNA is not accessible and it is bound and occluded by a nucleosome instead. This could reflect that the regulation of promoter accessibility by PRC1 is only effective in a subset of molecules within the population and thus in some cases RNAPII can access and bind polycomb-target promoters.

In conclusion, we have developed a free and amenable bioinformatic tool to extract single-molecule information of genome-wide NOMe-seq and BS-seq datasets generated using bulk populations. Complementarily, NOMePlot can be used to explore NOMe-sanger and BS-sanger sequences and facilitate their analysis and representation. As a use case we have analysed NOMe-assays data generated in mESCs using NOMePlot and have identified that bivalent promoters can be found in at least two alternative nucleosomal arrangement configurations that might have important implications to understand polycomb repression and pluripotency.

## Methods

### Coding of NOMePlot

NOMePlot is a user-friendly web application developed using R/shiny and other packages from Bioconductor repository. The input data in fasta format of Sanger sequencing is read by seqinr R package. In the case of High-throughput sequencing, the reference sequence is obtained from the available genome assembly at UCSC. The BAM file is read with Rsamtools. During the CpG/GpC analysis, the package Biostrings is used in both types of sequencing in order to read, align and estimate methylation levels of sequences. The package stats is used during the clustering of sequences. The heatmap and line plot of the sanger sequencing section are obtained by a sliding window algorithm, which calculate the density of methylation with a length specified by the user. The output data can be visualized from multiple ways, the graphical plots (heatmaps and line plots) are obtained with the package ggplot2, the genome browser is created with Gviz, the lollipop graphics are developed with the package grid. Finally, information reports are generated with the package Rmarkdown.

### Software implementation

NOMePlot can be used without knowledge of the R programming language. As calculations are performed at the server-side, the user does not need to consume local resources. In addition, installation is not required and thus the user will just need a web browser. To minimize misfunctioning due to long upload times, the current remote implementation of NOMePlot accepts bam files up to 50 MB which will cover genomic regions in the order of megabases with optimal coverage (around 1–2 million reads). NOMePlot is supported by most popular browsers and operative systems, and can be run on commodity computers, such as low-memory laptops. NOMePlot source code is available at GitHub to download and run locally with unlimited control of input file size. Typical processing times to analyse 400 bp genomic interval using file containing a genomic region of 500 000 bps with sequence coverage of 228X (1.5 Million 76 bp reads, size of 15 MB) is around 7 seconds and 14 seconds for the official shiny server (https://www.shinyapps.io) (8 GB dedicated RAM) and at local (i5 processor and 8 GB RAM) versions respectively.

### Input and output data

NOMePlot uses .fasta files as input for the Sanger sequencing analyses and .bam files as input for the high throughput sequencing module. An .html file is generated with a summary of results containing plots that can be opened with any web browser and easily converted into .png image files by dragging images to the desktop. A .csv dataset file containing the percentage of methylated CpGs or GpCs for all detected dinucleotides is generated and can be easily imported into a spreadsheet software to produce average methylation graphics. Lollipop graphics can be downloaded as .svg files that can be imported and edited in a vectorial graphic design software like Adobe Illustrator or Inkscape. A technical report is also generated in .html.

### Software availability

NOMePlot is freely accessible at http://www.landeiralab.ugr.es/software. Guided examples to learn how to use application are available at the welcome screen. The Shiny source code for local installation and modification is  available from GitHub at https://github.com/frequena/nomeplot under the GNU General Public License version 3.

### Single locus nucleosome occupancy and DNA methylation plots

NOMePlot generates .png files with average nucleosome occupancy and DNA methylation as connected scatter plots. Csv files containing the percentage of methylation at CpG or GpC sites were generated by NOMePlot and imported into Microsoft Excel to generate scatter plots with a trend line (polynomial order 6). Lollipop graphics were generated using a GpC clustering window of 140 and nucleosome positions were calculated using a region size length of 140 bp and a stringency value of 0.7.

### Cell culture

Mouse embryonic stem cells (background 129/Sv/C57BL/C6)^36^ were cultured in 5% CO_2_ at 37 °C on 0.1% gelatin-coated dishes DMEM KO (Gibco) media supplemented with 10% FCS, leukaemia-inhibiting factor (LIF), penicillin/streptomycin (Gibco), L-glutamine (Gibco) and 2-mercaptoethanol (Gibco) as described previously^37^.

### NOMe assay

Nuclei extraction and M.CviPI treatment were performed as described previously^15^. Briefly, isolated nuclei were resuspended in 1x reaction buffer containing 200 U of M.CviPI (NEB) and incubated for 15 min at 37 °C. For NOMe-PCR, bisulfite conversion was carried out using the EZ DNA methylation Kit (Zymo Research) according to the manufactures’ instructions. Amplified and purified DNA was cloned using pGEM-T easy kit (Promega). Primers used for PCR amplification are detailed in Table 1.

**Table 1:** Primers used in NOMe-PCR assay.

Actb	GAGTGATTTTTTGTTTATTTAATT
	TTCTAAATAATCCTCAAAACCCT
Msx1	GAGAAATTGTGGAAAGAAAGTAGTT
	AAAACAAAATCCTCCACTTTAACAC
Myf5	ATTAGTATATAAATTGATTTAATTTTTTGG
	TAAAACTATCTCTCTATAATTAACAAAAAC
Oct4	GGGGTGAGAGGATTTTGAAGGTTGAAAATGAA
	AACATAAAAAAATCCCCAATACCTCTAA
Pax3	GAATTTTTTGTGTTTTTTTTTAAATTT
	AAAAAAATCCCTTAAATACAAAATCCCA
Sox7	GGAGTTTTGGTGGAGTTTGGTTTTA
	TTTCCAAACTCTTATCCCCTAAAAAT
Tuba3a	TTTGATATTATAGGGTAAATTGAG
	CCTCCCCCAATAATCTTATCACTTA
Wnt1	GGGTTTTTGGGTGAGGAAGTGTTTTTA
	ACTAATCAAAACCACAAACAATAAATA

NOMe-seq libraries were prepared from 1 µg of DNA using the SureSelectXT Methyl-seq library prep kit (Agilent technologies) as described in manufactures’ instructions. Bisulfite conversion was performed using EZ DNA Methylation Gold kit (Zymo Research). Library was sequenced on Illumina NextSeq 500 using a 150-bp paired-end cartridge (17 million reads). Reads were aligned to the reference mouse genome mm9 to obtain the bam files using Bwa-meth 0.10^38^. Bam files were split into individual chromosomes using SAMtools and uploaded to NOMePlot to analyze individual molecules at single loci.

To obtain average nucleosome occupancy and DNA methylation plots for sets of genes (transcriptionally active and DNA methylated), we calculated CpG and GpC methylation counts (10 bp bins) using BisSNP 0.82.2^39^ followed by aaRon R package (http://github.com/astatham/aaRon) published elsewhere^15^. Scatter plots were generated using Microsoft Excel.

### Data access

NOMe-seq dataset of mESCs is available at GEO-NCBI with accession number GSE122964.

## Supplementary information

Supplementary figures
