# Supplementary material for: NOMePlot: analysis of DNA methylation and nucleosome occupancy at the single molecule
Source: Sci Rep. 2019 May 31;9:8140. doi: 10.1038/s41598-019-44597-2 (PMC6544651; doi:10.1038/s41598-019-44597-2)
Supplement: Supplementary file 1 — Supplementary figures [file 41598_2019_44597_MOESM1_ESM.pdf]

Supplementary information

NOMePlot: analysis of DNA methylation and nucleosome occupancy at the single molecule

Francisco Requena[1,2,3], Helena G. Asenjo[1,2,3], Guillermo Barturen[1], Jordi Martorell-Marugán[1], Pedro Carmona-Sáez[1] and David Landeira[1,2]

[1] Centre for Genomics and Oncological Research (GENYO), Avenue de la Ilustración 114, 18016 Granada, Spain

[2] Department of Biochemistry and Molecular Biology II, Faculty of Pharmacy, University of Granada, Granada, Spain

[3] Joined-first authors

Correspondence to David Landeira: davidlandeira@ugr.es

Running title: NOMePlot: Single molecule analysis of NOMe data

Keywords: Nucleosome binding, epigenetics, polycomb, embryonic stem cells, NOMe-Seq.

Contents:

Figure S1

Figure S2

Figure S3

Figure S4

A

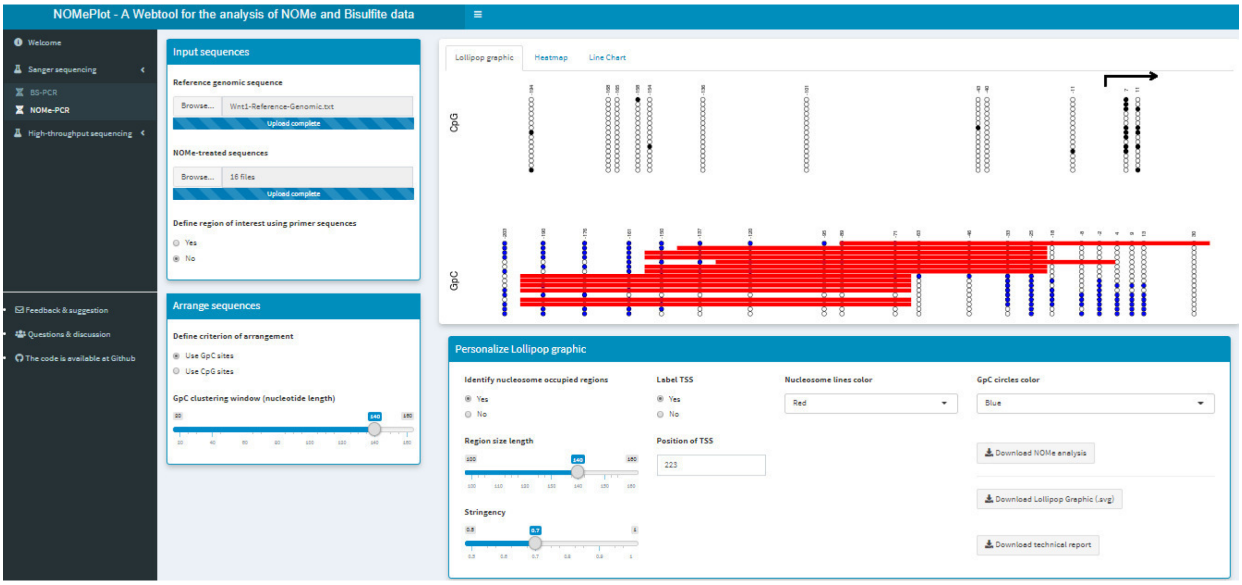

B

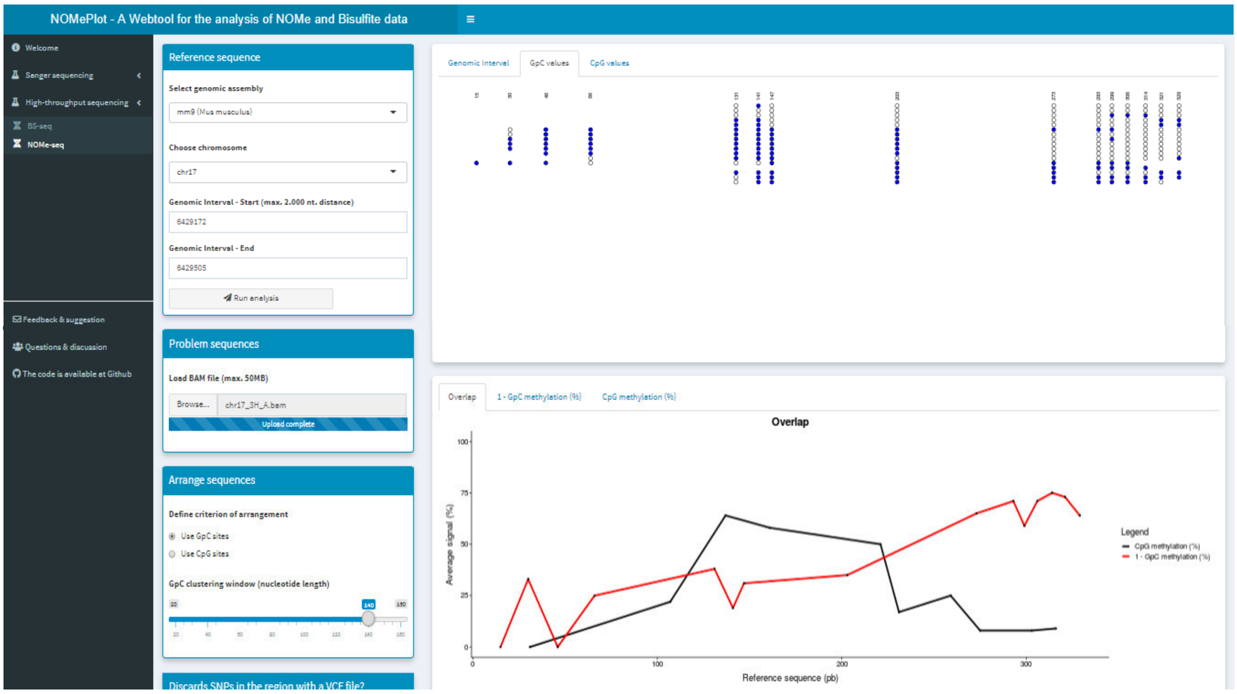

Figure S1. Screenshots of NOMePlot interface.

(A) Screenshot of NOMePlot app in Sanger sequencing mode for the analysis of NOMe-PCR. The image shows a lollipop graphic analysis being carried out and controls to change key parameters (i.e. clustering window, identification of occupied regions and stringency).

(B) Screenshot of NOMePlot app in high-throughput sequencing mode. Example shows a lollipop representation for GpC methylation for the genomic coordinates 6429172 to 6429505 of mouse chromosome 17 corresponding to *Dynlt1b* gene. The overlap diagram represents population-averaged signal for nucleosome occupancy (red line) and DNA methylation (black line) for the selected genomic region.

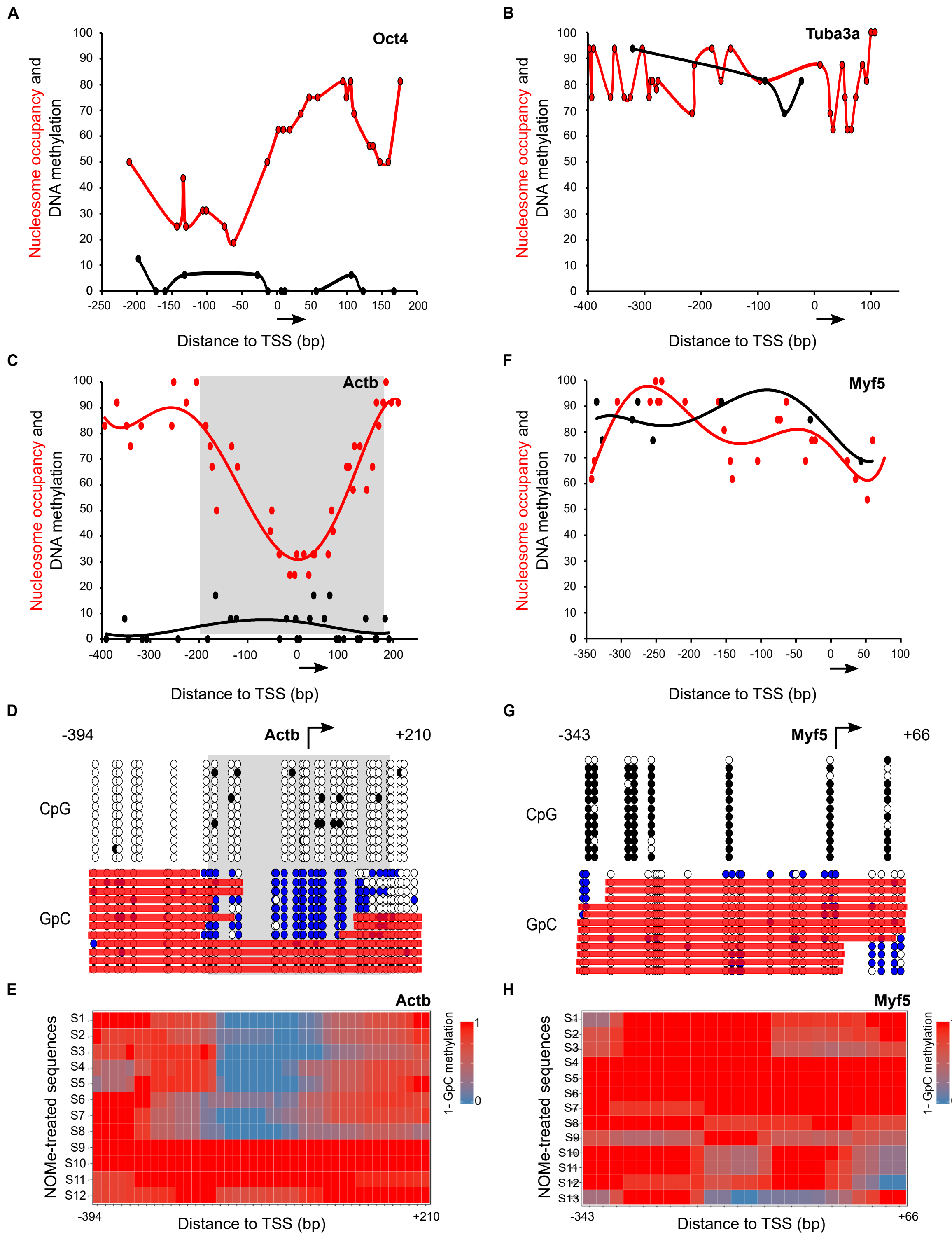

Figure S2. Analysis of *Actb* and *Myf5* gene promoters using NOMe-PCR followed by NOMePlot in mESCs.
(A-B) Connected scatter plots showing population-averaged nucleosome occupancy (red) and DNA methylation (black) relative to the TSS of an active gene (*Oct4*) (A) and a DNA methylated silent gene (*Tuba3a*) (B). Nucleosome occupancy is calculated as the percentage of unmethylated GpC sites. DNA methylation is plotted as the percentage of methylated CpG dinucleotides. Arrow indicates the direction of transcription.
(C, F) Scatter plots with trendline showing population-averaged nucleosome occupancy (red) and DNA methylation (black) relative to the TSS of an active gene (*Actb*) (C) and a DNA methylated silent gene (*Myf5*) (F). Nucleosome occupancy is calculated as the % of unmethylated GpC sites. DNA methylation corresponds to % of methylated CpG dinucleotides. Arrow indicates the direction of transcription.
(D, G) Lollipop diagram showing DNA methylation and nucleosome occupancy at the single molecule for *Actb* (D) and *Myf5* (G). Top panels show cytosine methylation at CpGs (white for unmethylated, black for methylated) and bottom panels show methylation patterns at GpC dinucleotides (white for unmethylated, blue for methylated). Red bars highlight GpC unmethylated regions long enough to accommodate a nucleosome. NDRs are marked with a grey box.
(E, H) Heatmap of nucleosome occupancy for *Actb* (E) and *Myf5* (H) upon clustering of twelve and thirteen sequences respectively. Nucleosome occupancy was calculated for each position around the TSS (X axis) as the average value of unmethylated cytosines at GpCs found within 140 bp windows. Color code goes from red (100 % occupancy, 1-GpG=1) to blue (0% occupancy, 1-GpC=0).

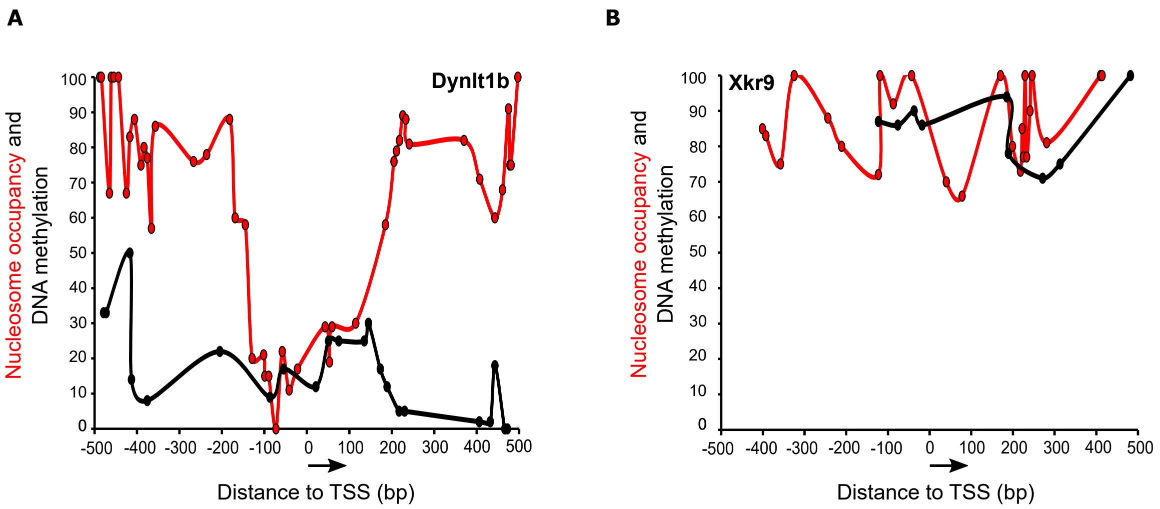

Figure S3. Locus-specific analysis of using NOMe-seq followed by NOMePlot.

(A-B) Connected scatter plots showing population-averaged nucleosome occupancy (red) and DNA methylation (black) relative to the TSS of an active gene (*Dynlt1b*) (A) and a DNA methylated silent gene (*Xkr9*) (B). Nucleosome occupancy is calculated as the percentage of unmethylated GpC sites. DNA methylation is plotted as the percentage of methylated CpG dinucleotides. Arrow indicates the direction of transcription.

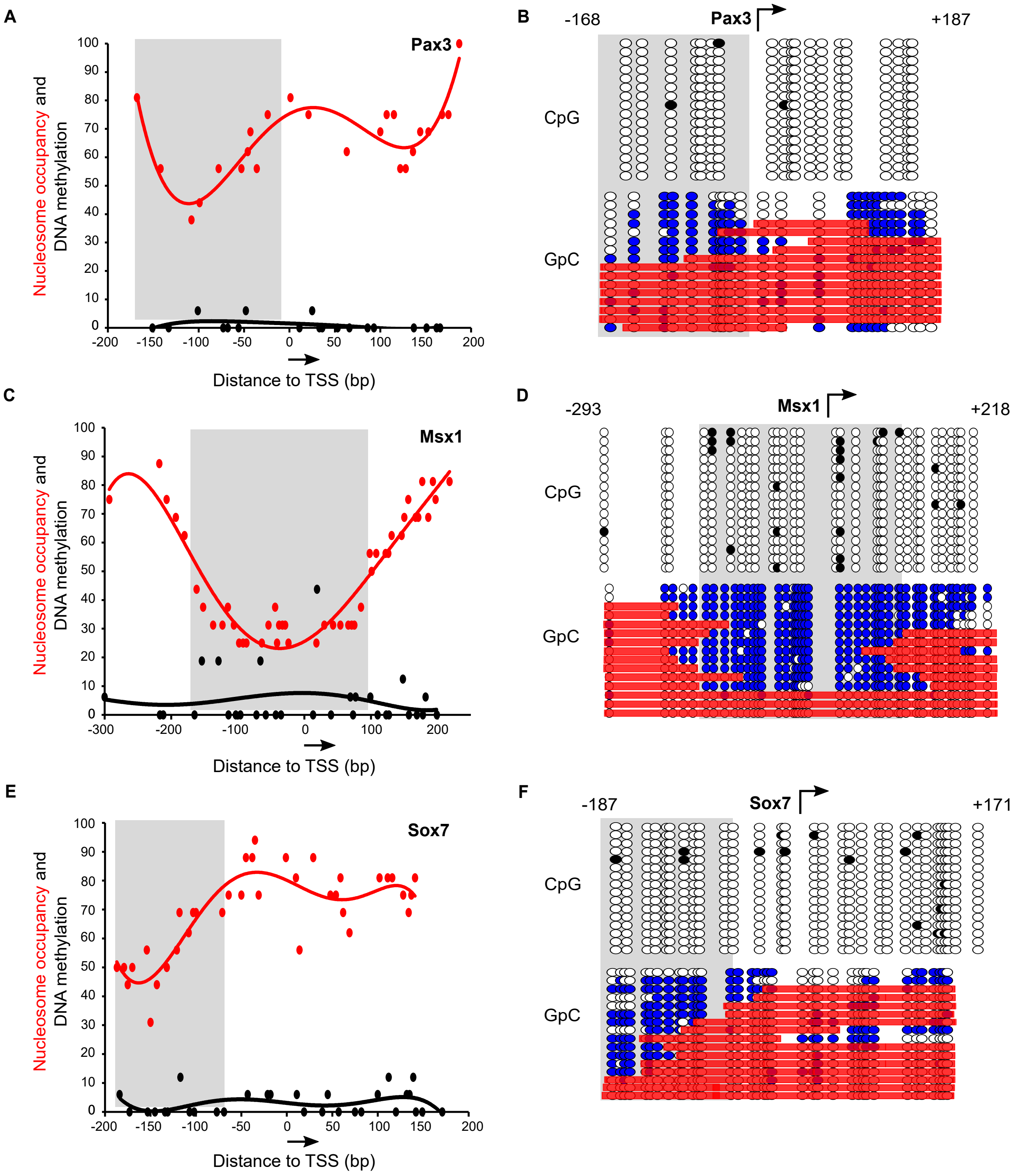

Figure S4. Analysis of nucleosome binding by NOMe reveals heterogeneous nucleosome arrangement at bivalent genes in mESCs.

(A, C, E) Scatter plots with trendline showing population-averaged nucleosome occupancy (red) and DNA methylation (black) relative to the TSS for a subset of bivalent genes (*Pax3*, *Msx1* and *Sox7*) analyzed by NOMe-PCR. Nucleosome occupancy is calculated as the percentage of unmethylated GpC sites. DNA methylation is plotted as the percentage of methylated CpG dinucleotides. Arrow indicates the direction of transcription.

(B, D, F) Lollipop diagram showing DNA methylation and nucleosome occupancy at the single molecule for *Pax3*, *Msx1* and *Sox7*. Top panels show cytosine methylation at CpGs (white for unmethylated, black for methylated) and bottom panels show methylation patterns at GpC dinucleotides (white for unmethylated, blue for methylated). Each line represents one molecule and each row correspond to a genomic position around the TSS (arrow). Red bars highlight GpC unmethylated regions long enough to accommodate a nucleosome. NDRs are marked with a grey box.
